# Salicine in Otorrhœa

**Published:** 1876-06

**Authors:** 


### SALICINE IN OTORRHŒA.

E. H. Jackson, M.D., of Lancaster, Ohio, says, in *Medical and Surgical Reporter:*

"Every physician is aware tnat the success attending the treatment of ulceration of the ears, or of chrcnic otorrhœa, is not very flattering, varied as the resources may be. With myself anything but a justifiable result in most cases was obtained, until I chanced to adopt salicine and calcined magnesia in combination. To the former of these I attribute the curative power, though the latter is an excellent therapeutical adjuvant.

"My experience with these remedies has been considerable, and I have yet to see the first case devoid of benefit. Of course there are some ear cases (as in all other classes of diseases) that cannot be relieved by any remedies so-called, but I am persuaded that by these means they are made to decrease in numbers. My method of treatment is as follows: —Ascertain the difficulty, its extent, nature, and state, either by natural or artificial examination, preferably the latter, *i. e.*, by the otoscope, speculum (Wilde's), mirror, etc. By these means you are better able to begin treatment intelligently. Prior to each examination and application syringe the ear well with tepid water; this may be soapy or clear; it should be soft water. Exercise care in this, as there is danger in undue pressure of the water upon the ear as it leaves the syringe. Place the syringe so that regurgitation may be unobstructed, and yet so that the water may freely reach the interior. After the ear is thoroughly cleansed and a speculum adjusted, blow into it through a quill—

R. Salicine.................................. gr. ij.

Cal. magnesia.......................... gr. iv.

and insert a small piece of cotton. Should the discharge be excessively offensive, the cotton can be wet with chlorinated soda, which will tend to allay the fetor. This process should be renewed every two or three days, observing well the effect, and varying the proportions of the medicine as demanded. In general, constitutional treatment is unnecessary, unless the otorrhœa depends on some dyscrasia. Much good, in the above procedure, attends the use of the water injections, but it is only a modicum compared with the salicine and magnesia. I am satisfied patient, continued use of these means will meet the desires of many who have heretofore been disappointed."

